# Late Diagnosis of Langerhans Cell Histiocytosis by Skin Biopsy in a Lung Transplant Candidate Patient

**DOI:** 10.7759/cureus.55226

**Published:** 2024-02-29

**Authors:** Francisco R Klein, Julia Klein, Diego Otalora Lozano, Carlos Vigliano

**Affiliations:** 1 Critical Care Medicine, Favaloro University, Faculty of Medical Sciences, Buenos Aires, ARG; 2 Critical Care Medicine, Favaloro Foundation University Hospital, Buenos Aires, ARG; 3 Critical Care Medicine, Sheba Medical Center, Intensive Care Unit, Ramat Gan, ISR; 4 Pathology, Favaloro Foundation University Hospital, Buenos Aires, ARG; 5 Board of Science and Technology (Dirección de Ciencia y Técnica, DCT), Institute of Translational Medicine, Transplantation and Bioengineering (IMeTTyB) Favaloro University-National Scientific and Technical Research Council (CONICET), Buenos Aires, ARG

**Keywords:** langerhans cell histiocytosis, acute respiratory failure, vv-ecmo, lung transplantation, skin biopsy

## Abstract

We present the case of a lung transplant candidate under veno-venous membrane oxygenation assistance (VV ECMO) whose diagnosis of emphysema of undetermined etiology was redefined as Langerhans cell histiocytosis (LCH) due to a scalp skin biopsy performed years after the beginning of his respiratory symptoms. A 20-year-old patient started three years before his admission with progressive dyspnea leading to a diagnosis of bullous emphysema of undetermined cause, which evolved into respiratory failure and evaluation for bilateral lung transplant. Three years later, he developed bilateral pneumonia requiring mechanical ventilation. When refractory hypoxemia ensued, he had to be placed on VV ECMO. Under these conditions, he was transferred to our center and listed for a bilateral pulmonary transplantation. Forty-eight hours after admission, and due to intense polyuria, central diabetes insipidus was diagnosed. In this clinical context, the presence of cutaneous lesions on the scalp was reconsidered and biopsied under the presumption of possible LCH, with pathology analysis confirming the diagnosis. He continued to be assisted with VV ECMO for 66 more days as a bridge to transplantation, developing multi-organ failure and passing away before a donor organ was available. The diagnosis of LCH should be considered in any adult patient with bullous emphysema of undetermined cause. Given the possibility of early therapeutic interventions, the search for its clinical associations (e.g., diabetes insipidus and/or skin lesions) should be a systematic part of the etiologic workup. The availability of skin specimens to reach a diagnosis makes its thorough search an important part of the diagnostic approach.

## Introduction

Langerhans cell histiocytosis (LCH) is considered the most common histiocytic cell neoplasm [1], with an incidence of 4.6 per million per year in children under 15 years of age [2] and at least one to 1.5 cases per million in adults [3,4]. In children, lung involvement occurs in 7.3-16.4% [5,6], while lung involvement in adults is seen in 47% of patients [7]. In most cases with pulmonary involvement, LCH behaves as a benign disease with unifocal involvement, almost exclusively affecting patients with a smoking habit [8]. In a recent series of lung transplant patients, LCH was the etiologic diagnosis in 0.25-0.4% of cases [9,10].

## Case presentation

**Figure 1:**
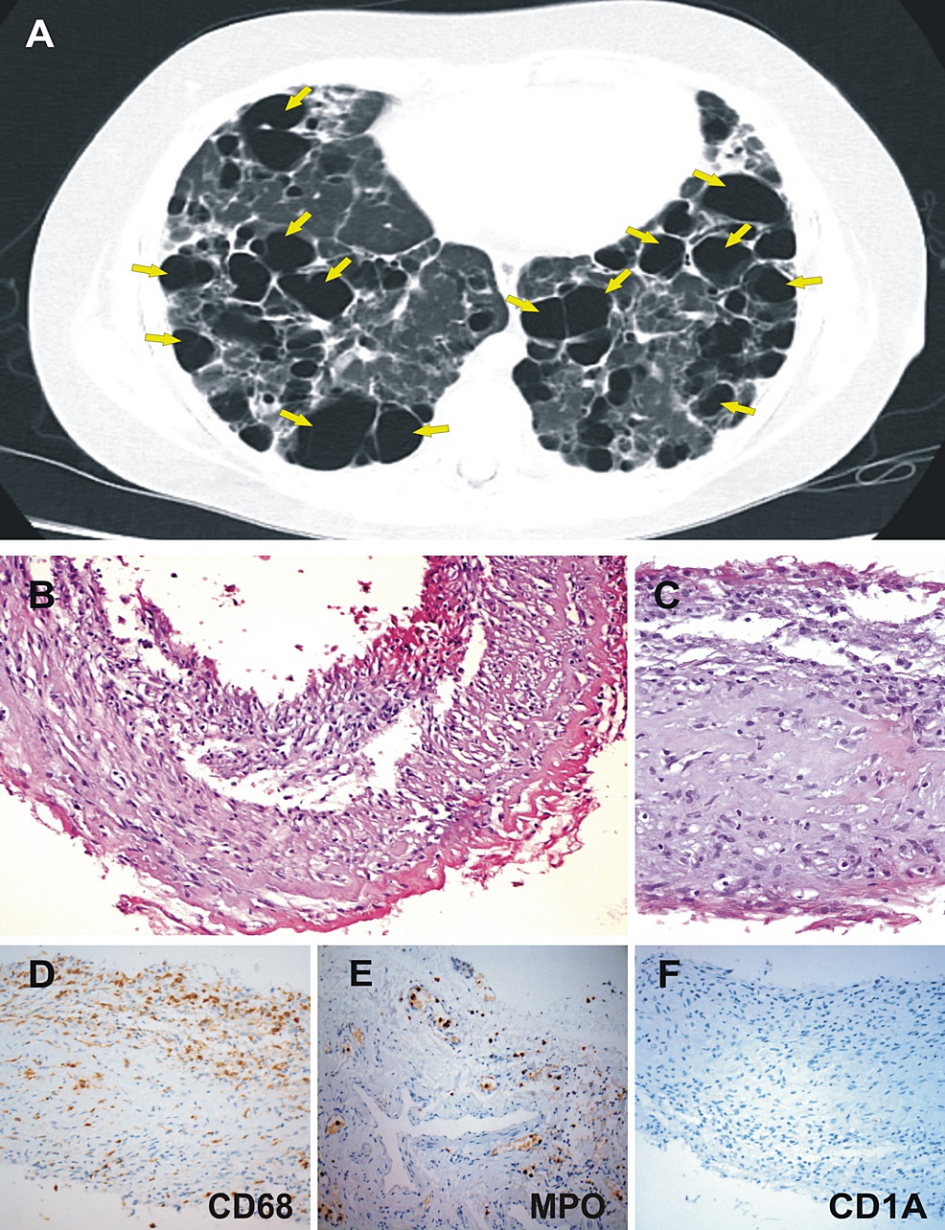
Chest tomography and pleural biopsy. (A) Chest computed tomography shows thin-walled cysts distributed symmetrically throughout both lungs (yellow arrows). (B-F) Videothorachoscopic obtained sample showed pleural tissue with fibrosis, vascular congestion, and mild inflammatory infiltrate (B) (H&E, x200); (C) H&E x400. A predominance of macrophages is shown in (D) (H&E x400) by CD68+ staining, with a regular number of polymorphonuclear cells as demonstrated by myeloperoxidase+ in (E) (H&E x400). Immunohistochemical determination for CD1a was negative (F) (H&E x400). These histological findings were considered as non-specific reactive pleural fibrosis.

A 20 year-old-patient, with no relevant clinical history for 17 years, started with cough and progressive dyspnea. A chest computed tomography (CT) scan performed at that time showed images suggestive of bullous emphysema and scarce bilateral interstitial pulmonary infiltrates leading to a presumptive diagnosis of bullous emphysema of undetermined cause. Over the next three years, dyspnea progressed to functional class IV (New York Heart Association) with worsening of the emphysematous lesions and the development of new and multiple bilateral and predominantly subpleural bullae (Figure 1A). 

A functional respiratory study performed at that time (36 months after his initial consultation) showed FEV1 = 19%, FVC = 28%, and FEV1/FVC: 88%. Plasma determinations of alpha-1 antitrypsin ruled out this deficit. During the same period, he developed lesions on the scalp that were interpreted as impetiginized seborrheic dermatitis and treated with oral clindamycin and topical corticosteroids that led to a partial improvement.

Given the rapid deterioration of his functional class, he was referred for transplant assessment and listed for a bilateral lung transplant. During a hospitalization 23 months prior to his last admission, he developed a left spontaneous pneumothorax leading to drainage, bullectomy, and subsequent videothoracoscopic chemical pleurodesis. A biopsy performed at that time reported pleural tissue with fibrosis, vasocongestion, and mild inflammatory infiltrate with few eosinophils and mononuclear predominance, which were considered nonspecific histological findings (Figure 1B-1F).

He remained in a rather stable condition for 11 months, under continuous home oxygen therapy with a 4.5 l/min flow, achieving a SaO_2_ of 90%. After this period, he was admitted with severe bilateral pneumonia, developing severe hypoxemia and hypercapnia and requiring mechanical ventilation. Unable to maintain adequate oxygenation, he soon required venovenous extracorporeal membrane circulation (VV-ECMO) support. Stabilized and transferred under VV-ECMO, he was admitted to the intensive care unit of our transplant center.

Since ICU admission, polyuria was another dominant sign. Hypernatremia (160 mEq/l), a urinary osmolality of 420 mOsm/kg, and a positive response to desmopressin favored the diagnosis of diabetes insipidus.

**Figure 2:**
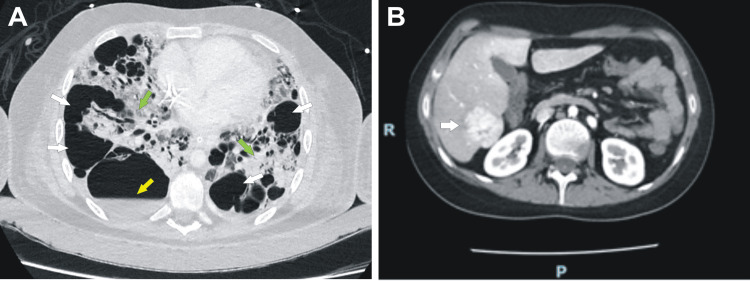
Chest and abdominal contrast-enhanced computed tomography. (A) Chest computed tomography showing progression of cystic lesions and bullae (white arrows), some of them with air-fluid levels (yellow arrow) coexisting with pulmonary infiltrates in the remaining parenchyma (green arrows). (B) Contrast-enhanced abdominal computed tomography showing a liver with a soft and regular contour and normal density. In segment VI, a 37 x 33 mm lesion was found (white arrow), showing an arterial-phase post-contrast enhancement.

The brain CT scan did not show any sellar or suprasellar lesions. Given the hemodynamic and respiratory instability, it was impossible to perform a brain MRI. Repeated transient interruptions of sedation and analgesia resulted in a normal neurological examination. A CT scan of the thorax showed the persistence of bullous images, some with hydroaerial levels, and the development of ground-glass infiltrates in the rest of the parenchyma with a tendency to consolidation (Figure 2A). A contrast-enhanced abdominal CAT scan demonstrated a nodular lesion in segment VI of the liver (Figure 2B).

**Figure 3:**
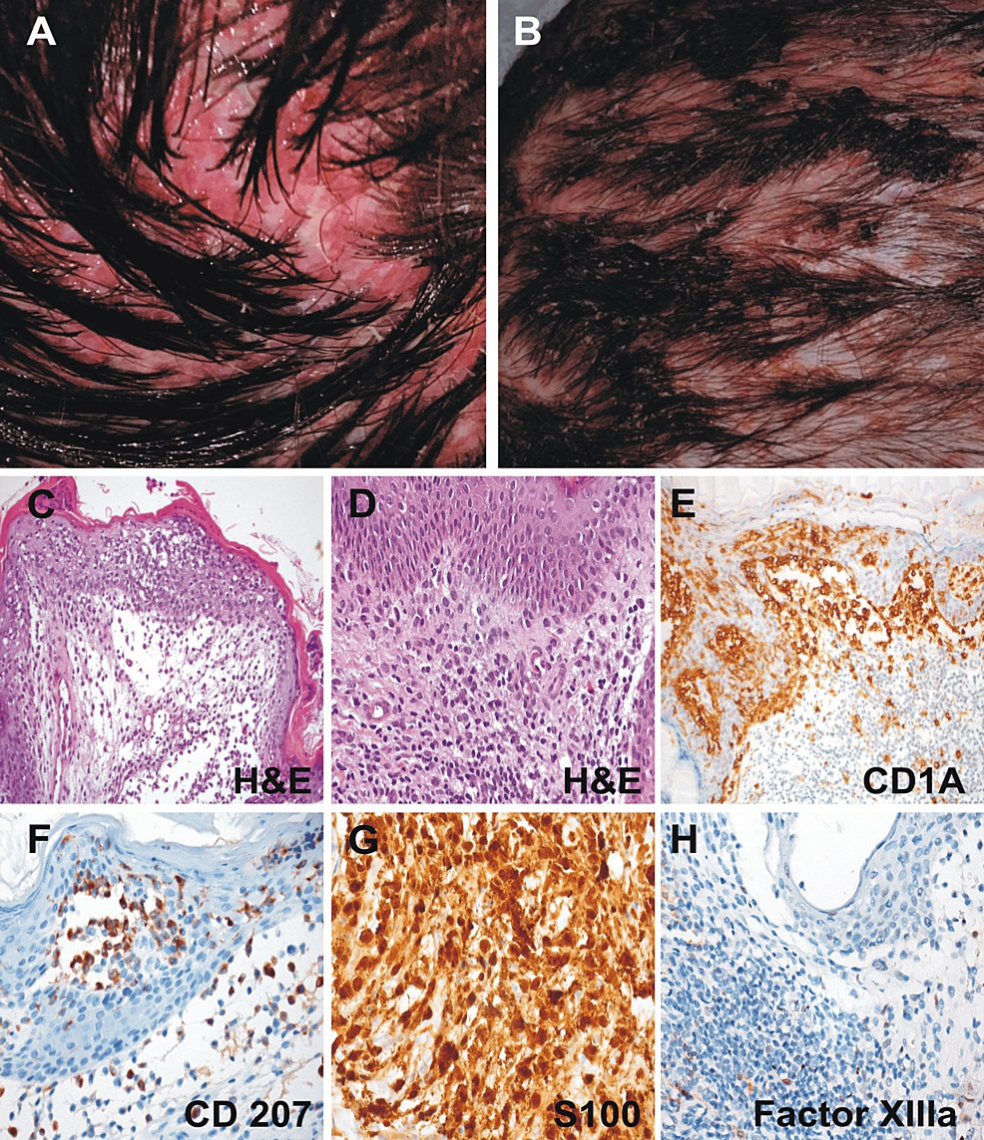
Scalp lesions: macroscopic and pathology features. (A, B) Physical examination revealed papulosquamous lesions with greasy scales affecting the scalp (A and B), predominantly in the occipital region, resembling seborrheic dermatitis. Biopsies were taken from such lesions for study. (C-H) This figure depicts a typical skin LCH lesion. Pathologic Langerhans cells with reniform nuclei and eosinophilic cytoplasm are scattered among an infiltrate of lymphocytes, eosinophils, and macrophages. The LCH cells react to immunostains for CD1a, CD207, and S100, but not for factor XIIIa. (C) H&E x200; (D) H&E x400; (E) CD1a x200; (F) CD207 x400; (G) S100 x400; (H) factor XIIIa x400.

Bronchoalveolar lavage (BAL) performed on days 2 and 15 of his admission to the ICU demonstrated nonspecific inflammatory cytologic findings. Physical examination at this point revealed papulosquamous lesions with greasy scales, affecting the scalp, resembling seborrheic eczema predominantly in the occipital region (Figure 3A-3B). Given the widespread pulmonary lesions, the development of diabetes insipidus and the presence of skin lesions were observed; on day 17, and under the presumption of LCH, a biopsy of the scalp lesions was taken and showed cell proliferation with positive immunohistochemistry for CD1a, S100, CD4, and CD68, with a Ki 67 proliferation index of 20%, confirming the histopathological diagnosis of this histiocytic neoplasm (Figure 3C-3H ).

The patient remained on VV-ECMO developing ventilator-associated pneumonia secondary to carbapenemase-producing *Pseudomonas aeruginosa *and persistent carbapenemase-producing *Klebsiella pneumoniae* bacteremia, with no documented primary focus. He developed progressive multiorgan failure and passed away before receiving a lung transplant after 66 days of ICU care.

**Figure 4:**
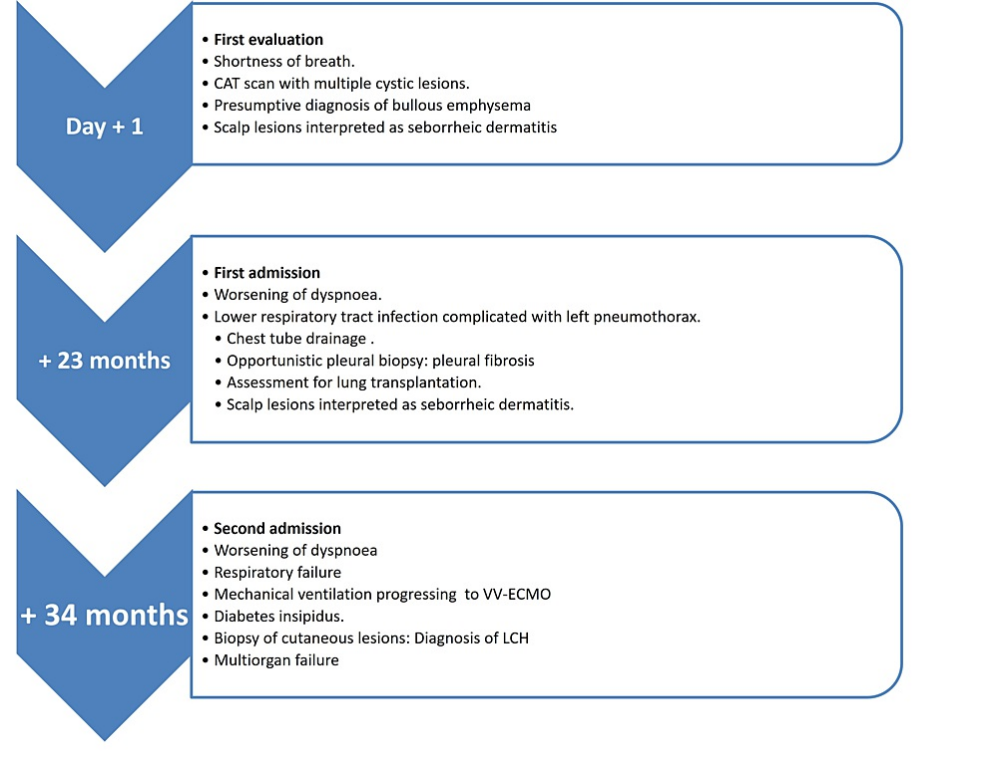
Timeline of the main hallmarks during the patient's clinical follow-up.

The main hallmarks in the patient's clinical follow-up are depicted in Figure 4.

## Discussion

The present report shows a case of LCH with pulmonary, cutaneous involvement and diabetes insipidus in a young adult in whom the diagnosis was made late and incidentally from the reconsideration of scarce pre-existing cutaneous lesions.

LCH is characterized by unregulated proliferation and differentiation from cells of myeloid origin [11]. In our case, immunohistochemical studies for CD68, S100, CD1a, and CD207 (Langerin) from skin biopsies confirmed the diagnosis although at a very advanced stage of the systemic disease. LCH can be classified according to the extent of the disease and organ involvement as unifocal or multifocal and monosystemic or multisystemic [4,7]. 

Multisystemic LCH is further subdivided into low-risk and high-risk LCH (the latter being defined as involving the liver, lungs, spleen, hematopoietic cells, or CNS) [12].

The reported case corresponds to a high-risk multifocal and multisystemic involvement with a clinical presentation where involvement of the skin, lungs, CNS (diabetes insipidus), and probably the liver was observed.

Hazim et al. in a retrospective study of adult patients with LCH found that of the 112 patients who initially presented with pulmonary signs and symptoms, only one had multisystem involvement [7]. The recommendations of the International Expert Consensus for the diagnosis and treatment of adult LCH recommend treatment with limited courses of chemotherapy [4]. There are two case reports of the use of ECMO in patients with multisystem LCH with pulmonary involvement (in one as a bridge to recovery [13] and in another as a bridge to lung transplantation [14], the former being diagnosed during ECMO as described in the case of our report). Both received chemotherapy treatment with favorable results. Given his critical condition, concomitant infections, and advanced disease, in the case we present, the patient was not a candidate for any chemotherapy regimen.

In a retrospective multicenter study of patients who underwent lung transplantation with a diagnosis of LCH, post-transplant recurrence rates of the disease were found to be 20% (usually responding favorably to chemotherapy treatment) [15], so the diagnosis of LCH should not be a contraindication for transplantation.

At the time of the initial diagnosis of emphysema of undetermined cause, which would later lead to respiratory failure, the few skin lesions that were previously interpreted and treated by a dermatologist as seborrheic dermatitis were not considered relevant for the diagnosis. In our patient, given the clinical stability achieved with VV ECMO, the etiological considerations of his bullous emphysema could be reconsidered, hierarchizing the recent diagnosis of diabetes insipidus and reformulating the meaning of the already known skin lesions. Definitive diagnosis was made possible by histopathological and immunohistochemical study of the skin lesions.

While isolated cutaneous manifestations are infrequent in adults diagnosed with LCH [16,17], in multisystem presentations of adults with LCH, cutaneous manifestations occur in up to 50% of cases [18,19,20]. 

This report illustrates the relevance of early consideration of alternative diagnoses in adult patients with bullous emphysema and/or spontaneous pneumothorax, including LCH. We emphasize the need for a careful dermatologic examination, given the high frequency of cutaneous involvement in adults with this condition.

## Conclusions

The high frequency of skin lesions in adults with multisystemic LCH makes it pertinent to systematically search for these manifestations in patients with clinical presentations compatible with the disease (emphysema of an undetermined cause, diabetes insipidus, hepatic lesions without etiological diagnosis, or others). Cutaneous LCH can be a great pretender, resembling a number of other common dermatoses, and may represent the earliest sign of the disease. The typical scalp lesions are small translucent papules, 1-2 mm in diameter, slightly raised, and rose-yellow in color. These lesions frequently show scaling or crusting, often leading to a misdiagnosis of seborrheic dermatitis. The accessibility of the pathological study of the skin samples would allow an early diagnosis and a potential treatment.
